# IL-1β/IL-6 signaling circuit in the tumor microenvironment drives prostate cancer development

**DOI:** 10.64898/2026.07.30.741572

**Published:** 2026-07-31

**Authors:** Young Sun Lee, Jimmy L. Zhao, Agnieszka Chryplewicz, Max Land, Roshan Sharma, Joseph Chan, Perianne Smith, Sanjay Kottapalli, Linda Fong, Zhenghao Chen, Eric Bent, HuiYong Zhao, Elisa de Stanchina, Wenfei Kang, Shevin Narine, Eric Rosiek, Ning Fan, Kayla Lawrence, Erolcan Sayar, Anuradha Gopalan, Ojasvi Chaudhary, Tianhao Xu, Ignas Masilionis, Ronan Chaligne, Michael Haffner, Rodrigo Romero, Dana Pe’er, Brett S. Carver, Charles L. Sawyers

## Abstract

Despite considerable progress in elucidating mechanisms leading to castration-resistant prostate cancer (CRPC), insight into the early stages of prostate cancer initiation and progression remains limited. Genomic drivers of prostate cancer initiation have been defined through patient tumor sequencing, but the subsequent events responsible for local tissue invasion are poorly understood. Here we leverage a well-studied genetically engineered mouse prostate cancer model (Hi-Myc) that, based on robust and reproducible kinetics for transitioning from pre-invasive prostatic intraepithelial neoplasia (PIN) to invasive prostate adenocarcinoma (PCa), provides an ideal system to systematically address this question using single-cell analysis. Surprisingly, the transcriptomic profiles of early PIN lesions are indistinguishable from those of late-stage, highly invasive tumor cells, suggesting that MYC activation at the PIN stage establishes a transcriptional program that is fully capable of driving invasion but is restrained by the local tumor microenvironment (TME). Indeed, we find that progression to PCa is associated with progressive infiltration of IL-1β ^+^ tumor-infiltrating macrophages at the PIN stage that, based on immunodepletion and cytokine neutralization experiments, are required for the PIN-to-PCa transition. Mechanistically, IL-1β from macrophages acts directly on prostate fibroblasts, leading to the release of IL-6, which drives invasion by activating IL-6R in tumor cells. Collectively, these findings identify a pro-tumorigenic IL-1β/IL-6 signaling circuit mediated through local macrophages and fibroblasts that unleashes the full oncogenic potential of a cancer driver (MYC) activated at the PIN stage. We also find evidence of this circuit in other (non-MYC-driven) prostate cancer models as well as human prostate and lung adenocarcinoma, with implications for TME-specific targeted therapeutics in early-stage disease.

## Full Text Availability

The license terms selected by the author(s) for this preprint version do not permit archiving in PMC. The full text is available from the preprint server.

